# Adjunctive Chinese Herbal Medicine therapy improves survival of patients with chronic myeloid leukemia: a nationwide population‐based cohort study

**DOI:** 10.1002/cam4.627

**Published:** 2016-01-15

**Authors:** Tom Fleischer, Tung‐Ti Chang, Jen‐Huai Chiang, Ching‐Mao Chang, Ching‐Yun Hsieh, Hung‐Rong Yen

**Affiliations:** ^1^ Graduate Institute of Chinese MedicineCollege of Chinese MedicineChina Medical UniversityTaichungTaiwan; ^2^ Department of Chinese MedicineChina Medical University HospitalTaichungTaiwan; ^3^ School of Chinese MedicineChina Medical UniversityTaichungTaiwan; ^4^ School of Post‐baccalaureate Chinese MedicineChina Medical UniversityTaichungTaiwan; ^5^ Management Office for Health DataChina Medical University HospitalTaichungTaiwan; ^6^ Graduate Institute of Integrated MedicineCollege of Chinese MedicineChina Medical UniversityTaichungTaiwan; ^7^ Research Center for Chinese Medicine and AcupunctureChina Medical UniversityTaichungTaiwan; ^8^ Center for Traditional MedicineTaipei Veterans General HospitalTaipeiTaiwan; ^9^ Graduate Institute of Clinical MedicineGraduate Institute of Traditional Chinese MedicineCollege of MedicineChang Gung UniversityTaoyuanTaiwan; ^10^ Division of Hematology and OncologyDepartment of Internal MedicineChina Medical University HospitalTaichungTaiwan; ^11^ Research Center for Traditional Chinese MedicineDepartment of Medical ResearchChina Medical University HospitalTaichungTaiwan

**Keywords:** Chinese herbal medicine, CML, leukemia, NHIRD, Taiwan

## Abstract

Despite good clinical results of current drugs, a good reason still exists to search for additional therapies for the management of Chronic Myeloid Leukemia (CML). Chinese Herbal Medicine (CHM) has thus far been overlooked by researchers and no data exists on the subject. We studied the impact of adjunctive CHM on the disease course of CML, using mortality as the major outcome measurement. We used the Taiwanese National Health Insurance Research Database to perform a nationwide population‐based cohort study. Our study included CML patients diagnosed between 2000 and 2010. We matched groups according to age, sex, Charlson Comorbidity Index (CCI) score and use of imatinib, and compared the Hazard Ratios (HR) of CHM group and non‐CHM users, as well as characterized trends of prescriptions used for treating CML. 1371 patients were diagnosed with CML in the years examined, of which 466 were included in to this study. We found that the HR of CHM group was significantly lower compared to non‐CHM groups (0.32, 95% CI 0.22–0.48, *P* < 0.0001). We also established that this association between reduced HR was dose‐dependent, and the longer CHM users received prescriptions, the lower the HR (*P* < 0.01). We also analyzed the most commonly used herbal products as well as the HR associated to their use, thus providing future research candidates. Our results supply a strong reason to assume that when administered by properly trained physicians, CHM may have a substantial positive impact on the management of CML.

## Introduction

First generation tyrosine kinase inhibitors (TKI) have revolutionized the treatment course of Chronic Myeloid Leukemia (CML) in the past decade and a half. Already in 2006, Imatinib was shown to aid 83% of patients diagnosed during the chronic phase of CML to reach a 5‐year event‐free survival 1. However, despite high initial response rates, this drug fails in up to 40% of cases due to resistance or side effects, and roughly 25% of initial major cytogenic response is lost by second year 2. In addition, population‐based studies revealed that the performance of imatinib in ‘real life’ do not match those achieved in trial settings 3. Data from our own institute confirms that under the right conditions, Taiwanese CML patients taking imatinib may indeed experience sustained remission similar to that achieved in follow‐ups of the IRIS 8 trials 4. However, it has been shown in the past that overall adherence to imatinib is very low in Taiwan. A study from 2012 noted that only 38.7% of patients continued imatinib for ≥18 months without interruption, and a mere 7.7% continued imatinib for ≥5 years 5.

In addition, the leap forward in the treatment of CML is still confined to the chronic phase. Median survival time of imatinib patients who eventually enter blast phase of CML is 7 months alone, and even Hematopoietic Stem Cell Transplant (HSCT) provides only limited improvement of survival time in these cases 6, 7, 8. All these urge researchers to continue seeking additional therapies.

Virtually no data exists regarding the use of Chinese Medicine (CM) for the treatment of CML. During the past 30 years, the number of CM clinical trials conducted in China have been on the rise, and reviews show that 11% of these are of leukemic disorders 9. Unfortunately, the standards upheld by these trials have not met requirements of peer‐reviewed journals, and thus past trials have failed to reach western readership. However, the existing studies from China as well as many written accounts from CM physicians point out that a deeper look into the matter is justified.

Chinese Medicine (CM) is commonly used in Asia. Data from 2001 shows that 28% of the Taiwanese population covered by the National Health Insurance (NHI) program had visited a CM outpatient at least once during that same year, and twice the amount of patients had sought CM during the entire timeframe analyzed in the study (1996–2001). In addition, the study suggested that at that time point, utilization of CM by patients in the NHI system was still rising 10. Ever since the implementation of the NHI system in 1995, the vast majority of Taiwanese residents have been registered in the program, and in 2012 more than 99% of the population in Taiwan was registered as beneficiaries 11. The Taiwanese NHI program has been reimbursing CM since 1996 and recognizes three major categories of treatment: (1) Chinese herbal products. These are either single‐herb or multi‐herb products which have been manufactured by Good Manufacturing Practice (GMP)‐certified pharmaceutical companies which ensure quality and consistency of product batches. (2) Acupuncture, which includes also moxibustion and cupping. (3) Manual therapy, including acupressure, Tuina, and chiropractic. Only treatments administered by licensed CM physicians are reimbursed by the NHI.

All treatments provided as part of the NHI are recorded in the Taiwanese NHI Research Database (NHIRD). CM entails different types of treatments, some of which are more difficult to accurately reproduce. Thus, we chose to focus on Chinese Herbal Medicine (CHM), which is provided to patients as part of the NHI program in granulated form. Single‐herb products are produced from concentrated extracts of one single herb (or parts of it), while multi‐herb products are a formula composed of several herbs. GMP companies monitor the consistency of batches through active ingredients.

We set out to make use of the data in the NHIRD to provide insight into the effects of CHM on the outcomes of patients diagnosed with CML.

## Methods

### Database

The NHIRD consists of records from all outpatient and hospitalization incidence of all its beneficiaries, as well as details of the administered treatment, and drugs or CHM prescribed. All diseases in the NHIRD are classified, using the Ninth Revision, Clinical Modification (ICD‐9‐CM). For this study, we collected patient information through the Registry for catastrophic illness patients file which included the entire NHI records of all CML patients in Taiwan. In order for patients to be registered in this file, their diagnosis must be approved by a qualified pathologist, ensuring the confirmation of disease. After which we searched these patients corresponding information, including comorbidities, in the NHIRD inpatient and outpatient files.

### Study population

We included all patients, aged 18 and above, diagnosed with CML (ICD‐9‐CM: 205.1), between January 2000 and December 2010, with follow‐up time defined as December 31, 2011. When the analyzing hazard ratio for Charlson Comorbidity Index (CCI) was used, it has been reported that a higher score on the Charlson comorbidity index was associated with decrease of up to 50% in overall‐ and event‐free survival 12. There is very little data associating between different comorbidities and general prognosis of CML patients, however we found no better alternative 13. This study was conducted in accordance with the Helsinki Declaration. To protect personal privacy, the electronic database was decoded for research with patient identification scrambled. According to National Health Research Institutes regulations, informed consent is not required because patient identification has been decoded. Therefore, our study was exempted from institutional review board approval of Public Health, Social and Behavioral Science Committee Research Ethics Committee, China Medical University and Hospital.

### Statistical analysis

SAS 9.4 (SAS Institute Inc., Cary, NC, USA) was used for statistical analysis of all information retrieved from the NHIRD. Chi‐square tests were performed to determine statistical significance for comparisons; *t*‐test was used for normally distributed variables and Fisher's exact test to compare catagorial variables. Exploratory analyses of Hazard Ratios (HR) were performed through a Cox proportional hazard model was used, taking in account age, gender, urbanization level, CCI score, and use of imatinib, with a 95% confidence interval (CI). Kaplan–Meier and log rank tests were used for categorical covariates. Only *P* values <0.05 were considered statistically significant.

### Network Analysis

The core patterns of CHMs used in treating CML patients were identified with an open‐sourced freeware NodeXL (http://nodexl.codeplex.com/), and all the selected two drugs combinations were applied in this network analysis. The line width, ranging from 1 to 5 in the network figure was defined by counts of connections between a CHM and co‐prescribed CHMs, and thicker width of line indicated significant prescription patterns. The network analysis manifested the top five core patterns of top 50 combinations in this survey.

## Results

**Figure 1:**
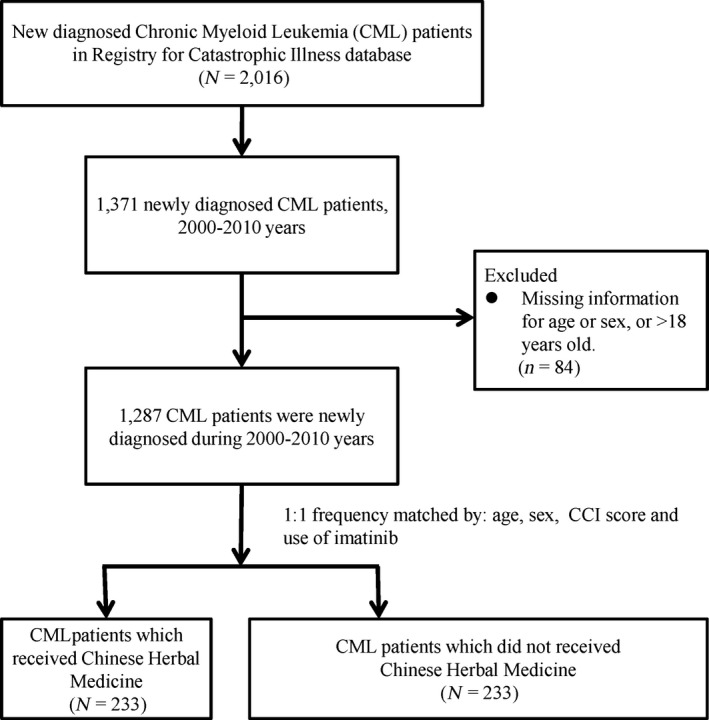
Study population flowchart diagram. Of the total amount of Chronic Myeloid Leukemia (CML) patients registered in the NHIRD (*n* = 2016), 1371 patients were diagnosed within the years 2000–2010. After excluding patients with missing information of age >18, as well as matching 1:1 by age, sex, Charlson Comorbidity Index (CCI), and use of imatinib, both groups contained 233 patients.

Of the 1371 Taiwanese patients diagnosed with CML between 2000–2010, after matching both groups for age, sex, CCI score and use of imatinib, both groups each contained *n* = 233 patients (Fig. 1.). The patients who received CHM treatment were registered as receiving Chinese medicine specifically for the diagnosis CML. Both groups presented similar characteristics, and there were no statistically significant differences (*P* < 0.05), other than a minor, however significant (*P* = 0.0489) difference in the use of busulfan (Table 1.).

**Table 1:** Characteristics of chronic myeloid leukemia patients according to use of CHM

	CHM				
	No (*N* = 233)		Yes (*N* = 233)		*P*‐value
	*n*	%	*n*	%	
Gender^3^					0.99
Female	77	33.05	77	33.05	
Male	156	66.95	156	66.95	
Age mean^4^ ± SD^3^ (years)	48.08 (16.18)		48.10 (16.15)		0.9909
Age group^3^					0.99
18–39	83	35.62	83	35.62	
40–59	94	40.34	94	40.34	
≥60	56	24.03	56	24.03	
Urbanization level^1^, ^3^					0.6389
1 (highest)	53	22.75	47	20.17	
2	70	30.04	79	33.91	
3	45	19.31	50	21.46	
4 (lowest)	65	27.9	57	24.46	
CCI score 3					0.99
0	164	70.39	164	70.39	
1	14	6.01	14	6.01	
≥2	55	23.61	55	23.61	
Drug					
Busulfan^5^	10	4.29	3	1.29	0.0489
Dasatinib^3^	31	13.3	21	9.01	0.1412
Hydroxyurea^3^	134	57.51	125	53.65	0.4014
Imatinib^3^	195	83.69	195	83.69	0.99
Interferon^3^	25	10.73	35	15.02	0.1666
Nilotinib^3^	25	10.73	26	11.16	0.882
Follow time (mean, median)^2^	3.44 (2.83)		4.39 (4.02)		

CHM included only Chinese Herbal Medicine, excluded acupuncture, and manual therapies.

CHM, Chinese Herbal Medicine; CCI, Charlson Comorbidity Index.

^1^The urbanization level was categorized by the population density of the residential area into 4 levels, with level 1 as the most urbanized and level 4 as the least urbanized.

^2^Cessation of follow time was defined as expiration or end of study timeframe.

^3^Chi‐Square Test, ^4^ *t*‐test, ^5^fisher‐exact test.

**Figure 2:**
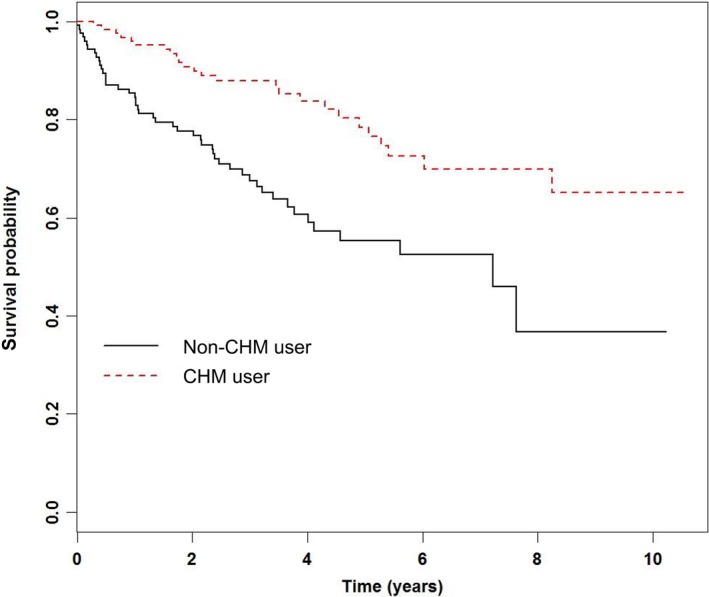
Kaplan–Meier plot of overall survival in patients with chronic myeloid leukemia, according to Chinese Medicine (CM). The curves were adjusted for the drugs: busulfan, dasatinib, hydroxyurea, imatinib, interferon, nilotinib, or any combination of these. The cohort contained patients who were registered as receiving CM but no standard drugs, as well as patients who received neither CM nor drugs, as shown in Table 3. These groups were much smaller in size, and were therefore excluded from this plot.

After adjusting for the use of CHM, age, gender, urbanization level, CCI score and drug use, the CHM group displayed a much lower mortality hazard ratio (HR) (0.32, 95% CI 0.22–0.48, *P* < 0.0001) when compared to the non‐CHM group (Table 2). The difference in mortality of the two groups was also illustrated through a Kaplan–Meier survival graph, as shown in Figure 2 (*P* < 0.0001).

**Table 2:** Cox model with hazard ratios and 95% confidence intervals of mortality associated with CHM and covariates among chronic myeloid leukemia patients

Variable	Number of deaths	Crude^1^			Adjusted^2^		
		HR	(95%CI)	*P*‐value	HR	(95%CI)	*P*‐value
CHM use (ref=non‐CHM users)							
No	86	1	reference		1	reference	
Yes	41	0.39	(0.27–0.57)	<.0001	0.32	(0.22–0.48)	<.0001
Age							
18–39	23	1	reference		1	reference	
40–59	42	1.7	(1.02–2.82)	0.0418	1.95	(1.14–3.33)	0.0143
≥60	62	5.37	(3.32–8.69)	<.0001	4.21	(2.37–7.48)	<.0001
Gender							
Male	34	1	reference		1	reference	
Female	93	0.71	(0.48–1.06)	0.0918	0.90	(0.59–1.35)	0.6020
Urbanization level							
1	18	1	reference		1	reference	
2	45	1.79	(1.03–3.09)	0.0381	1.45	(0.82–2.55)	0.1973
3	24	1.36	(0.74–2.51)	0.3195	1.29	(0.70–2.41)	0.4155
4 (lowest)	40	1.88	(1.08–3.27)	0.0265	1.39	(0.79–2.47)	0.2553
CCI score							
0	79	1	reference		1	reference	
1	7	1.12	(0.52–2.42)	0.7788	0.92	(0.42–2.05)	0.8409
2	41	1.59	(1.08–2.32)	0.0175	0.81	(0.52–1.25)	0.3328
Drug							
Busulfan	8	2.23	(1.09–4.47)	0.0281	0.92	(0.40–2.11)	0.8372
Dasatinib	11	0.71	(0.38–1.32)	0.274	1.06	(0.55–2.05)	0.8657
Hydroxyurea	103	2.96	(1.88–4.66)	<.0001	2.34	(1.46–3.76)	0.0004
Imatinib	78	0.23	(0.16–0.33)	<.0001	0.36	(0.22–0.57)	<.0001
Interferon	25	1.35	(0.87–2.11)	0.1853	1.48	(0.91–2.39)	0.1131
Nilotinib	6	0.38	(0.17–0.86)	0.0204	0.44	(0.19–1.04)	0.0618

CHM, Chinese Herbal Medicine; CCI, Charlson Comorbidity Index.

^1^Relative hazard ratio; ^2^adjusted hazard ratio, mutually adjusted for CHM use, age, gender, urbanization level, CCI score and imatinib use in Cox proportional hazard regression.

We then set out to test the impact of prolonged consumption of herbal medicine on mortality risk, and so we calculated HR according to the sum of days patients received prescriptions. Table 3 demonstrates that CHM users which accumulated 0–30 days of prescription (*n* = 135) had a reduction in HR compared to patients which did not receive CHM (HR 0.43, 95% CI 0.28–0.67, *P* < 0.001). Similarly patients who received 30–180 days of CHM (*n* = 73) had a further reduction of HR (0.25, 95% CI 0.13–0.46, *P* < 0.001), and patients who received >180 days (*n* = 25) had the lowest HR (0.7, 95% CI 0.1–0.53, *P* < 0.01).

**Table 3:** Hazard Ratios and 95% confidence intervals of mortality risk associated with cumulative use day of CHM among chronic myeloid leukemia patients

Number of CHM visits/per year	*N*	Number of deaths	Person years	IR	Crude HR	Adjusted HR^1^
					(95% CI)	(95% CI)
Non‐CHM users	233	86	800.706	107.41	1(reference)	1(reference)
Chinese herb users						
0–30 days	135	28	586.319	47.76	0.47 (0.31–0.72)***	0.43 (0.28–0.67)***
30–180 days	73	12	323.064	37.14	0.36 (0.20–0.65)***	0.25 (0.13–0.46)***
>180 days	25	1	113.509	8.81	0.08 (0.01–0.61)*	0.07 (0.01–0.53)**

IR, incidence rates per 1000 person‐years; HR, hazard ratio; CI, confidence interval.

^1^Adjusted HR represented adjusted hazard ratio: mutually adjusted for age, gender, urbanization level, CCI score and treatment in Cox proportional hazard regression.

*P<0.05; **P<0.01;***P < 0.001.

**Figure 3:**
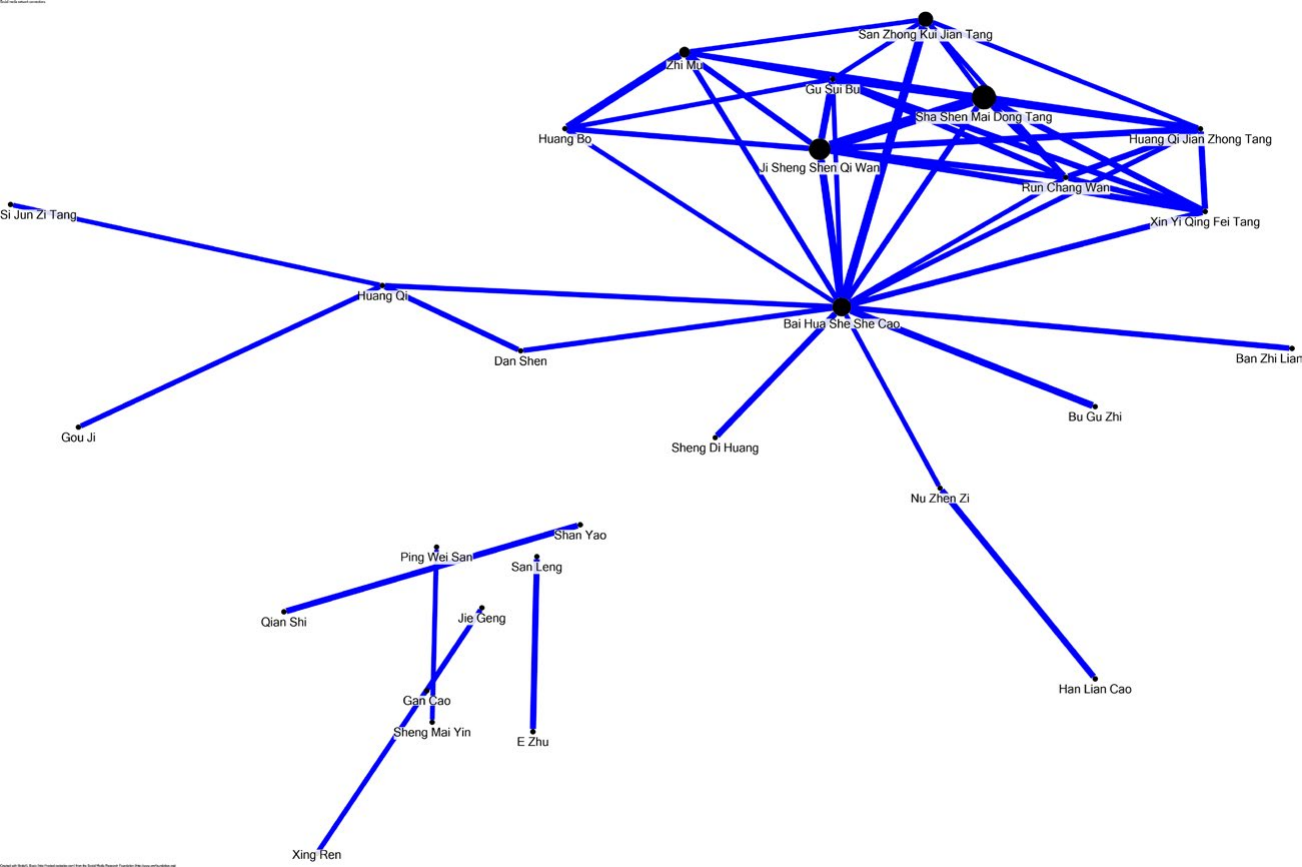
Network analysis of the top 50 multi‐herb and single‐herb products prescribed together for Chronic Myeloid Leukemia (CML) patients. Prescriptions were analyzed through open‐sourced freeware NodeXL and the core pattern of these CHMs showed that *Sha‐Shen‐Mai‐Dong‐Tang, Ji‐Sheng‐Shen‐Qi‐Wan, Bai‐Hua‐She‐She‐Cao, San‐Zhong‐Kui‐Jian‐Tang, and Zhi‐Mu* are among the most frequently used combinations.

In Table 4 are listed the ten most commonly prescribed single‐herb and multi‐herb products for the treatment of CML, as well their respective HR. It may be seen in Table 4 that of the twenty products, only four did not achieve a *P* value <0.05. In addition, we presented the average amounts these products were prescribed per visit (Table 5.) as well as the common combinations physicians utilized in prescriptions (Fig. 3.)

**Table 4:** Hazard Ratios and 95% confidence intervals of mortality risk associated with cumulative use of single herbs and herbal formulas among chronic lymphocytic leukemia patient

				Hazard Ratio(95% CI)	
		*n*	Frequency of mortality	Crude^1^	Adjusted^2^
*Non‐Chinese Herbal Medicine group*				1(reference)	1(reference)
Single‐herb products					
Pin yin nomenclature	Scientific name				
Bai Hua She She Cao	*Hedyotis diffusa*	9	1	0.29 (0.04–2.08)	0.33 (0.05–2.46)
Dan Shen	*Saliva miltiorrhiza*	30	5	0.39 (0.16–0.97)*	0.26 (0.10–0.65)**
Huang Qi	*Astragalus membranaceus*	29	8	0.61 (0.29–1.26)	0.33 (0.15–0.73)**
Shan Yao	*Dioscorea opposita*	12	3	0.58 (0.18–1.84)	0.28 (0.09–0.93)*
Sheng Di Huang	*Rehmannia glutinosa*	25	4	0.38 (0.14–1.05)	0.24 (0.09–0.71)**
Gan Cao	*Glycyrrhiza glabra*	35	6	0.39 (0.17–0.90)*	0.32 (0.14–0.74)**
Yan Hu Suo	*Corydalis yanhusuo*	44	8	0.38 (0.18–0.78)**	0.26 (0.12–0.56)***
Ji Xue Teng	*Spatholobus suberectus*	22	1	0.11 (0.02–0.77)*	0.08 (0.01–0.55)*
Sha Ren	*Amomum villosum*	28	7	0.59 (0.27–1.28)	0.48 (0.22–1.08)
Mai Men Dong	*Ophiopogon japonicus*	30	4	0.30 (0.11–0.81)*	0.23 (0.08–0.64)**
Multi‐herb products					
Pin yin nomenclature	Scientific name				
Ji Sheng Shen Qi Wan	–	17	2	0.25 (0.06–1.00)	0.19 (0.05–0.81)*
Ping Wei San	–	26	3	0.24 (0.08–0.77)*	0.22 (0.07–0.72)*
Jia Wei Xiao Yao San	–	28	1	0.08 (0.01–0.55)*	0.10 (0.01–0.73)*
Shen Ling Bai Zhu San	–	21	4	0.39 (0.14–1.05)	0.30 (0.11–0.83)*
Qi Ju Di Huang Wan	–	13	2	0.34 (0.08–1.38)	0.28 (0.07–1.15)
Shao Yao Gan Cao Tang	–	41	5	0.25 (0.10–0.62)**	0.19 (0.08–0.48)***
Sheng Mai Yin	–	26	7	0.59 (0.27–1.27)	0.34 (0.15–0.76)**
Gui Pi Tang	–	21	6	0.68 (0.30–1.57)	0.60 (0.25–1.43)
Liu Wei Di Huang Wan	–	30	5	0.32 (0.13–0.78)*	0.19 (0.07–0.48)***
Xin Yi Qing Fei Tang	–	18	1	0.12 (0.02–0.84)*	0.12 (0.02–0.89)*

Crude HR^1^ represented relative hazard ratio; Adjusted HR^2^ represented adjusted hazard ratio: mutually adjusted for age group, gender, urbanization level, number of comorbidity, and drug used in Cox proportional hazard regression.

*P < 0.05,**P < 0.01,***P < 0.001.

**Table 5:**
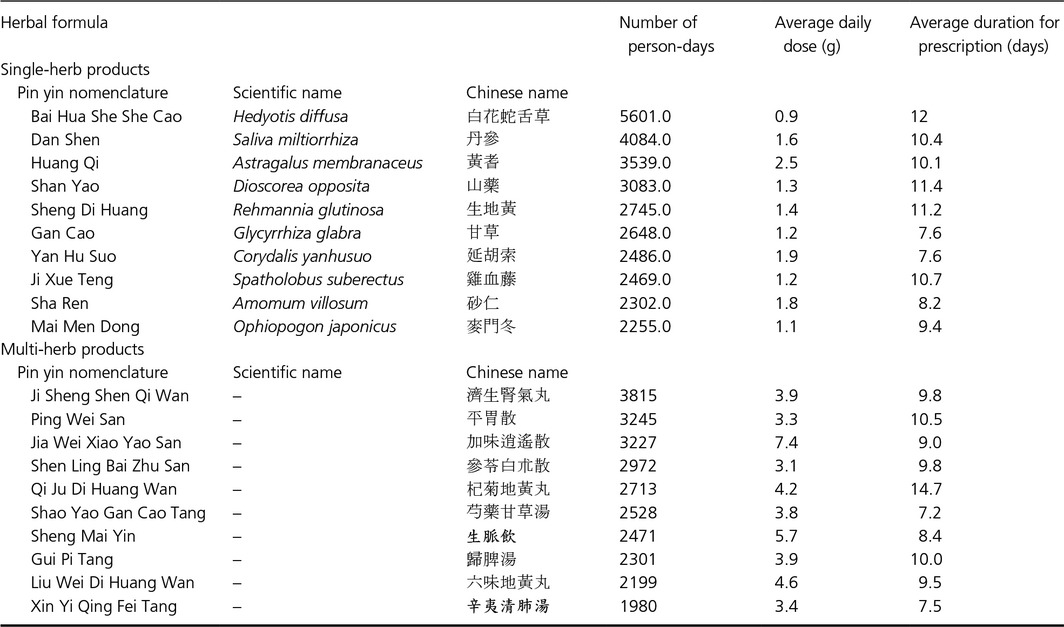
Ten most common single herbs and herbal formulas prescribed

## Disscussion

There are virtually no data regarding the use of any form of complementary alternative medicine (CAM), for the treatment of CML. This might partially be due to the relative scarcity of CML. In the United States, for example, the annual incidence of newly diagnosed cases range from 1.0 to 1.3 per 100,000 14 and data regarding CAM for the more prevalent chronic lymphoid leukemia, is slightly more available 15, 16. During the course of our study, we revealed that CHM was an essential part of therapy, and less than 2% of the CM group received acupuncture alone, which was part of our motivation to limit the scope of the study to CHM alone. This is important to notice as acupuncture has become the more prevalent modality of CM in most Western countries. In both England and Australia, for example, the number of registered acupuncturists far outnumber that of CHM practitioners 17, 18. In the recent *American National Health Interview Survey,* acupuncture was ranked the 11th most frequently used CAM, while CHM did not even exist as a category in the survey 19.

Our most significant finding was the lower risk of mortality associated with the utilization of CHM among CML patients (HR 0.32, 95% CI 0.22–0.48, *P* < 0.0001), as shown in Table 2. This improvement in survival can also be seen in the Kaplan–Meier curve, which clearly illustrates that patients in the CHM group had longer survival time. In addition, it was shown that this improvement was dose dependant, and the longer patients received CHM, the lower their HR was (Table 3).

A relatively short survival times was analyzed in this study (3.44 years for non‐CHM group, 4.39 years for CHM group, Table 1). At first, this finding raised our suspicion since studies suggest that the vast majority of patients in Taiwan are diagnosed during the chronic phase, and therefore survival time should be as long as that recorded in western countries 5, 20, 21. However, a recent study published by Chen et al. using a smaller cohort, and with a study timeframe equal to our own, reported comparable follow‐up times 22.

One limitation of our study may be clarified through this same paper, namely, the influence of adherence to imatinib on survival time. As described in the introduction, adherence to imatinib among Taiwanese patients is far from ideal. One of our concerns was that higher motivated patients would potentially be more adherent to drug therapy, which would undoubtedly affect survival, as shown by Chen et al. as well 22. Due to the nature of a retrospective study, we were unable to rule out differences in motivation among the two groups. Instead, we analyzed the data again matching the two groups according to the average amount of days‐per‐year imatinib was prescribed. Sample size was decreased by nearly half (both groups had *n* = 124 patients) and average sum of days‐per‐year imatinib was prescribed was 230 days (Table S1, Fig. S1). After matching, HR of CHM group was still lower compared with non‐CHM group, *P* value <0.0001 (Table S2). A dose‐dependent decrease in HR, in conjunction with the fact that CHM group presented lower HR even after matching to “imatinib days”, does not substantiate causal relation between CHM and better survival. Nonetheless, these findings challenge the notion that improved survival was merely due to patients' motivation.

An additional limitation of this study is that certain prognostic factors which are associated with poor prognosis of CML, are not available through the NHIRD, namely: age, spleen size, high platelet count, presence of circulating blasts, marked basophilia or eosinophilia, and the presence of additional chromosomal abnormalities (double Ph, 8 + , 17q+) are all 23. We were thus unable to rule out the possibility that the differences in HR between the two groups stemmed from these factors.

Equally important, this study provides future research candidates in form of specific herbal products. It is beyond the scope of this paper to discuss the potential therapeutic effects of each product, though it should noted that while some of the products found, example *Hedyotis diffusa,* have already been researched in the context of leukemia, other products are novel findings and provide candidates for studies in this field.

Although modern treatments have truly transformed CML into a chronic disease, there is clearly reason to keep on searching for additional therapies. The data supplied here is from a relatively large population, spanning over a significant length of time. Our results supply reason to assume that adjunctive CHM may have a strongly positive impact on the management of CML. More research is required to substantiate if this is true to non‐Asian patients as well, and clinical trials would need to be conducted in order to confirm the causal relation between the CHM and the outcomes displayed.

## Conflict of Interest

None declared.

## Supporting information


**Figure S1.** The sum of days imatinib was prescribed per year, on average for non‐Chinese Herbal Medicine (CHM) group, as well as CHM group.
**Table S1.** Characteristics of chronic myeloid leukemia patients matched by drug‐day‐per‐year, according to use of Chinese Herbal Medicine (CHM)
**Table S2.** Cox model with hazard ratios and 95% confidence intervals of mortality associated with Chinese Herbal Medicine (CHM) and covariates among chronic myeloid leukemia patients.
