# Supplementary material for: Adjunctive Chinese Herbal Medicine therapy improves survival of patients with chronic myeloid leukemia: a nationwide population‐based cohort study
Source: Cancer Med. 2016 Jan 15;5(4):640–8. doi: 10.1002/cam4.627 (PMC4831282; doi:10.1002/cam4.627)
Supplement: Supplementary file 1 — Figure S1. The sum of days imatinib was prescribed per year, on average for non‐Chinese Herbal Medicine (CHM) group, as well as CHM group. Table S1. Characteristics of chronic myeloid leukemia patients matched by drug‐day‐per‐year, according to use of Chinese Herbal Medicine (CHM) Table S2. Cox model with hazard ratios and 95% confidence intervals of mortality associated with Chinese Herbal Medicine (CHM) and covariates among chronic myeloid leukemia patients. [file CAM4-5-640-s001.docx]

As already mentioned in the main article, we were aware of the fact that adherence to TKI’s in Taiwan has been far from ideal, and one statistical artifact we were keen on challenging was: supposing Chinese Herbal Medicine (CM) patients had higher motivation to stay fit, their adherence to TKI therapy was likely to be better as well, thus affecting survival time.

We were unable to directly examine drug adherence. Instead, we examined the amount of prescriptions patients received. Since imatinib is a 1^st^ line TKI, and the most-commonly prescribed in our cohort as well, we looked into the sum of the prescription days-per-year of imatinib. We discovered that imatinib was in-fact prescribed for a longer duration on average in the CHM group, by roughly 10%.

In order to test the influence of this factor, we re-analyzed the data and matched the two groups according to the amount of days-per-year of imatinib prescriptions. After matching, there were n=124 patients in each group (Supp 3.) and both received on average 230 days of imatinib per-year (Supp 4.). Hazard ratio of CHM group remained lower when compared to non-CHM group (Supp 5.). Examining imatinib prescriptions is not equivalent to examining adherence, and in addition some discontinuations of imatinib are due to reason such as HSCT or death, (see Chang et al. 2012, main article reference). However, matching “imatinib days” does contribute additional input for the interpretation of these results.

Since HR did not increase, we decided to use the original results with the larger sample size in the main article.

| Table S1. Characteristics of chronic myeloid leukemia patients matched by drug-day-per-year, according to use of Chinese Herbal Medicine (CHM) | | | | | | | | |
| --- | --- | --- | --- | --- | --- | --- | --- | --- |
|  |  | CHM | | | | |  | *p*-value |
|  |  | No (N =124) | |  | Yes (N =124) | |  |  |
|  |  | n | % |  | n | % |  |  |
| Gender |  |  |  |  |  |  |  | 0.99 |
| Female |  | 41 | 33.06 |  | 41 | 33.06 |  |  |
| Male |  | 83 | 66.94 |  | 83 | 66.94 |  |  |
| Age mean±SD ^a^ ( years) |  | 50.42 (16.69) | |  | 50.66 (16.58) | |  | 0.9107 |
| Age group |  |  |  |  |  |  |  | 0.99 |
| 18-39 |  | 38 | 30.65 |  | 38 | 30.65 |  |  |
| 40-59 |  | 46 | 37.1 |  | 46 | 37.1 |  |  |
| ≥60 |  | 40 | 32.26 |  | 40 | 32.26 |  |  |
| Urbanization level |  |  |  |  |  |  |  | 0.6536 |
| 1 (highest) |  | 31 | 25 |  | 23 | 18.55 |  |  |
| 2 |  | 37 | 29.84 |  | 41 | 33.06 |  |  |
| 3 |  | 24 | 19.35 |  | 24 | 19.35 |  |  |
| 4 (lowest) |  | 32 | 25.81 |  | 36 | 29.03 |  |  |
| CCI score |  |  |  |  |  |  |  | 0.99 |
| 0 |  | 82 | 66.13 |  | 82 | 66.13 |  |  |
| 1 |  | 5 | 4.03 |  | 5 | 4.03 |  |  |
| ≥2 |  | 37 | 29.84 |  | 37 | 29.84 |  |  |
| Drug |  |  |  |  |  |  |  |  |
| Busulfan |  | 4 | 3.23 |  | 3 | 2.42 |  | 0.7014 |
| Dasatinib |  | 4 | 3.23 |  | 8 | 6.45 |  | 0.2365 |
| Hydroxyurea |  | 64 | 51.61 |  | 61 | 49.19 |  | 0.7032 |
| Imatinib |  | 86 | 69.35 |  | 86 | 69.35 |  | 0.99 |
| Drug day/per year (mean±SD)^a^ |  | 229.80 (80.94) | |  | 230.36 (82.10) | |  | 0.964 |
| Interferon |  | 13 | 10.48 |  | 11 | 8.87 |  | 0.6675 |
| Nilotinib |  | 7 | 5.65 |  | 5 | 4.03 |  | 0.554 |
| Follow time (mean, median) |  | 3.18 (2.81) | |  | 4.23 (3.72) | |  |  |
| Chi-Square Test, ^a^ t-test | | | | | | | | |
| ^†^: The urbanization level was categorized by the population density of the residential area into 4 levels, with level 1 as the most urbanized and level 4 as the least urbanized. | | | | | | | | |
|  | | | | | | | | |


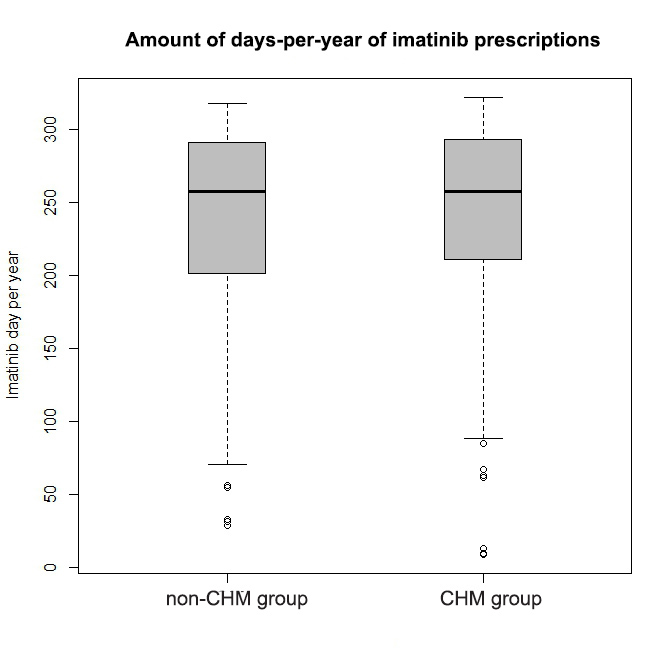


Figure S1. The sum of days imatinib was prescribed per year, on average for non-Chinese Herbal Medicine (CHM) group, as well as CHM group.

| Table S2.. Cox model with hazard ratios and 95% confidence intervals of mortality associated with Chinese Herbal Medicine (CHM) and covariates among chronic myeloid leukemia patients | | | | | | | | | |
| --- | --- | --- | --- | --- | --- | --- | --- | --- | --- |
|  | No. of die |  | Crude^*^ | | |  | Adjusted^†^ | | |
| Variable |  |  | HR | (95%CI) | p-value |  | HR | (95%CI) | p-value |
| CHM use (ref=non-CHM users) |  |  |  |  |  |  |  |  |  |
| No | 48 |  | 1.00 | reference |  |  | 1.00 | reference |  |
| Yes | 25 |  | 0.41 | (0.25-0.67) | 0.0003 |  | 0.27 | (0.16-0.45) | <.0001 |
| Age |  |  |  |  |  |  |  |  |  |
| 18-39 | 10 |  | 1.00 | reference |  |  | 1.00 | reference |  |
| 40-59 | 16 |  | 1.34 | (0.61-2.94) | 0.4729 |  | 1.56 | (0.65-3.71) | 0.3169 |
| ≥60 | 47 |  | 6.13 | (3.09-12.17) | <.0001 |  | 3.62 | (1.5-8.77) | 0.0043 |
| Sex |  |  |  |  |  |  |  |  |  |
| male | 21 |  | 1.00 | reference |  |  | 1.00 | reference |  |
| female | 52 |  | 0.72 | (0.43-1.21) | 0.2199 |  | 1.11 | (0.63-1.95) | 0.7265 |
| Urbanization level |  |  |  |  |  |  |  |  |  |
| 1 | 14 |  | 1.00 | reference |  |  | 1.00 | reference |  |
| 2 | 24 |  | 1.37 | (0.7-2.66) | 0.3581 |  | 1.01 | (0.49-2.08) | 0.985 |
| 3 | 14 |  | 1.13 | (0.54-2.37) | 0.7476 |  | 0.89 | (0.4-1.97) | 0.7703 |
| 4 (lowest) | 21 |  | 1.28 | (0.65-2.52) | 0.4766 |  | 0.82 | (0.4-1.67) | 0.5782 |
| CCI score |  |  |  |  |  |  |  |  |  |
| 0 | 42 |  | 1.00 | reference |  |  | 1.00 | reference |  |
| 1 | 3 |  | 1.10 | (0.34-3.56) | 0.8718 |  | 1.21 | (0.34-4.33) | 0.7738 |
| 2 | 28 |  | 1.55 | (0.95-2.52) | 0.0768 |  | 0.67 | (0.38-1.19) | 0.1701 |
| Drug |  |  |  |  |  |  |  |  |  |
| Busulfan | 5 |  | 2.02 | (0.81-5) | 0.131 |  | 0.63 | (0.2-1.99) | 0.4277 |
| Dasatinib | 3 |  | 0.74 | (0.23-2.35) | 0.6102 |  | 2.72 | (0.75-9.8) | 0.1264 |
| Hydroxyurea | 58 |  | 3.65 | (2.03-6.56) | <.0001 |  | 2.32 | (1.21-4.47) | 0.0116 |
| Imatinib | 24 |  | 0.16 | (0.1-0.26) | <.0001 |  | 0.16 | (0.08-0.33) | <.0001 |
| Interferon | 10 |  | 1.38 | (0.7-2.73) | 0.3492 |  | 1.22 | (0.53-2.81) | 0.6375 |
| Nilotinib | 3 |  | 0.80 | (0.25-2.53) | 0.6978 |  | 1.79 | (0.52-6.19) | 0.3548 |
| Crude HR^＊^ represented relative hazard ratio; Adjusted HR^†^ represented adjusted hazard ratio: mutually adjusted for CHM use, age, gender, urbanization level, CCI score, drug use and treatment in Cox proportional hazard regression. | | | | | | | | | |
